# Public support for neonatal screening for Pompe disease, a broad-phenotype condition

**DOI:** 10.1186/1750-1172-7-15

**Published:** 2012-03-14

**Authors:** Stephanie Shifra Weinreich, Tessel Rigter, Carla Geertruida van El, Wybo Jan Dondorp, Pieter Johannes Kostense, Ans T van der Ploeg, Arnold JJ Reuser, Martina Cornelia Cornel, Marloes Louise Catharina Hagemans

**Affiliations:** 1 Department of Clinical Genetics and Center for Lysosomal and Metabolic Diseases, Erasmus MC University Medical Center, Rotterdam, The Netherlands; 2 Department of Clinical Genetics/EMGO Institute for Health and Care Research, VU University Medical Center, Amsterdam, The Netherlands; 3 Department of Health, Ethics and Society, Faculty of Health, Medicine and Life Sciences, Research Institutes GROW and CAPHRI, Maastricht University, Maastricht, The Netherlands; 4 Department of Epidemiology and Biostatistics, VU University Medical Center, Amsterdam, The Netherlands; 5 Department of Pediatrics, Division of Metabolic Diseases and Genetics and Center for Lysosomal and Metabolic Diseases, Erasmus MC University Medical Center, Rotterdam, The Netherlands; 6 Department of Clinical Genetics/EMGO Institute for Health and Care Research, VU University Medical Center, Postbox 7057, BS7 H459, Amsterdam 1007 MB, Netherlands

**Keywords:** Neonatal screening, Glycogen storage disease type II, Technology assessment, Biomedical, Health policy, Consumer participation

## Abstract

**Background:**

Neonatal screening for Pompe disease has been introduced in Taiwan and a few U.S. states, while other jurisdictions including some European countries are piloting or considering this screening. First-tier screening flags both classic infantile and late-onset Pompe disease, which challenges current screening criteria. Previously, advocacy groups have sometimes supported expanded neonatal screening more than professional experts, while neutral citizens' views were unknown. This study aimed to measure support for neonatal screening for Pompe disease in the general public and to compare it to support among (parents of) patients with this condition. The study was done in the Netherlands, where newborns are not currently screened for Pompe disease. Newborn screening is not mandatory in the Netherlands but current uptake is almost universal.

**Methods:**

A consumer panel (neutral group) and (parents of) patients with Pompe disease (Pompe group) were sent information and a questionnaire. Responses were analyzed of 555 neutral and 58 Pompe-experienced informants who had demonstrated sufficient understanding.

**Results:**

87% of the neutral group and 88% of the Pompe group supported the introduction of screening (95% CI of difference -10 to 7%). The groups were similar in their moral reasoning about screening and acceptance of false positives, but the Pompe-experienced group expected greater benefit from neonatal detection of late-onset disease. Multivariate regression analysis controlling for demographics confirmed that approval of the introduction of screening was independent of having (a child with) Pompe disease. Furthermore, respondents with university education, regardless of whether they have (a child with) Pompe disease, were more likely to be reluctant about the introduction of screening than those with less education, OR for approval 0.29 (95% CI 0.18 to 0.49, p < 0.001).

**Conclusions:**

This survey suggests a rather high level of support for newborn screening for Pompe disease, not only among those who have personal experience of the disease but also among the general public in the Netherlands. Optional screening on the basis of informed parental consent is probably unrealistic, underlining the need for new guidelines to help policymakers in their consideration of newborn screening for broad phenotype conditions.

## Background

New treatments as well as high-throughput and multiplex screening methods are stimulating policymakers and legislators to consider adding new diseases to blood-based neonatal screening panels. An internationally accepted goal of neonatal screening is benefit for the neonate [1]. Lately, neonatal screening policy discussion is also addressing the value of genetic knowledge for family members besides the infant [2] and the value of a predictive diagnosis without the (immediate) possibility or need for intervention [3]. Several lysosomal storage disorders are being considered in the context of expanded neonatal screening (reviewed in [4]), including Pompe disease.

Pompe disease (MIM ID #232300) is an autosomal recessive enzyme deficiency due to mutations in the gene coding for acid alpha-glucosidase (*GAA*; MIM ID *606800). Insufficient alpha-glucosidase activity leads to accumulation of glycogen in the cells and eventually to progressive muscle weakness. Pompe disease has a broad geno- and phenotypic spectrum. The most severe, classic infantile form of disease has a birth prevalence of about 1:138,000 in the Netherlands [5]. It presents at a median age of 1.6 months but is usually diagnosed between 4.5 and 5.3 months [6]. The natural course of classic infantile disease includes cardiac hypertrophy and rapidly progressive muscle weakness; without treatment, infants rarely survive beyond 1 year of age [7]. Pre- and post-marketing studies taken together show that enzyme replacement therapy has a positive effect on the heart, prevention of muscle weakness and life expectancy, especially when treatment starts early. However, some children with classic infantile Pompe disease do not get long-term benefit from enzyme replacement therapy, even when it is given promptly. One predictive factor is CRIM status (cross-reactive immunological material), which reflects whether patients produce any endogenous acid alpha-glucosidase [8,9].

Besides the classic infantile form of Pompe disease, there is a milder, more slowly progressive form often referred to as late-onset which affects about 1:57,000 people [5]. It is often referred to as late-onset Pompe disease and this term will be used in this paper, though it must be stressed that this slowly progressive form can manifest at almost any age, from infancy through the fifth decade, often with a considerable delay between first complaints and diagnosis [10]. The heart is not affected but there is progressive, proximal muscle weakness and often respiratory problems. Many patients become dependent on assisted ventilation and need a wheelchair. An 18-month randomized, controlled trial showed a modest but significant positive effect of enzyme replacement therapy on the ability to walk and on the stabilization of pulmonary function [11]. This and earlier studies suggest that the better the condition of the patient at start of therapy, the greater the benefit. Late-onset patients currently receiving therapy may have been diagnosed decades before therapy was available. Therefore the full potential of therapy will not be known until greater numbers of patients have started treatment soon after diagnosis. A separate question is how best to shorten the delay between first complaints and diagnosis.

The first large-scale experience of neonatal screening for Pompe disease, including clinical follow-up, comes from Taiwan. Over 300,000 newborns have been screened using a fluorimetric assay and evolving diagnostic algorithms which include the relative amount of acarbose-resistant acid alpha glucosidase [12-14]. Full performance statistics have not been published since the pilot phase of the program when false positive rates were rather high [12]. Clinical benefit of neonatal screening has been reported for classic infantile cases (all CRIM positive) [13]. The screening and diagnostic algorithms in Taiwan have so far led to identification of 13 infants without cardiac involvement, who have been classified with 'later-onset' Pompe disease. They were put under surveillance, and some have started treatment with enzyme replacement therapy [14]. Pilot studies without clinical follow-up have been done using various techniques, in Japan [15], Austria [16] the United States [17] and northern Germany [18]. At the time of writing, four U.S. states have mandated screening for Pompe disease to start by 2012 [4].

Currently published first-tier screening methods using blood spots cannot distinguish between classic infantile and late-onset Pompe disease. Confirmatory testing, which includes clinical and laboratory procedures, will identify classic infantile cases but will alert even more parents to the possibility of late-onset disease in their child. Although a severity-rating scale for *GAA *mutations has been developed [19], genotyping offers little certainty in predicting the age of symptom onset and the rate of disease progression for late-onset patients [20,21]. Thus some infants flagged by screening will become "patients-in-waiting" [22,23]. The ethical complexity of neonatal screening for Pompe disease has indeed been recognized in the literature [24-28]. Neonatal screening for Pompe disease will detect more than twice as many late-onset cases as classic infantile cases; in fact it is likely that the proportion of late-onset cases is currently underestimated, as neonatal screening for other lysosomal storage disorders has identified larger than expected numbers of (probable) late-onset cases [29-31]. This prospect raises the question of whether the expected benefits of neonatal screening for Pompe disease do indeed outweigh its possible drawbacks. This issue of 'proportionality' is especially important if the screen falls under a directive paradigm of protection through public health. It might be less problematic under a paradigm of individual choice by consumers. Although previous studies have addressed parental acceptability of neonatal screening for a treatable late-onset disease [32,33] or a hypothetical, untreatable late-onset disease, [34] there is a lack of studies which address acceptability of neonatal screening for a treatable disease of broad phenotype such as Pompe disease.

The aims of this study were to measure support for neonatal screening for Pompe disease in the general public and to test whether (parents of) patients differ from the general public in their support for neonatal screening for their condition. A questionnaire specific to neonatal screening for Pompe disease was developed. The study aimed at a broad age range of informants, in contrast to previous studies on hypothetical acceptability of expanded neonatal screening in the general population which targeted prospective parents or parents with children under 18 [34,35].

A broad age base was sought because neonatal screening for Pompe disease touches on long-term predictive testing, an emerging field which may affect people in many phases of life. The questionnaire was grounded in descriptive ethics [36]. Screening can be studied as a public health issue (should the state implement it for the benefit of the population?) or as a matter of individual choice (does the informed consumer want to use the test?). In this study, informants were asked to think along both perspectives. This approach is relevant for jurisdictions like the Netherlands where although newborn screening is a routine procedure with high uptake, it is not mandatory. The main outcome measures were (1) informed judgement on whether the government should offer neonatal screening for Pompe disease and (2) informed, hypothetical choice to use the screening. The size of the respondent groups permitted exploration of a few extra demographic variables which might explain the main outcome measures. Educational level was chosen because it has been shown previously that lower educational level is associated with stronger willingness to extend neonatal screening criteria [35]. Gender was also included because male respondents are underrepresented in many surveys on issues in neonatal screening e.g. [35,37]. The survey included two other major features. It explored the moral reasoning for the main outcome measures, focussing on ethical principles which often appear in discourse on neonatal screening frameworks. Finally, the survey offset benefits and harms of neonatal screening for Pompe disease in case of a positive first-tier blood spot test.

## Methods

### Overview of study design

Using a newly developed questionnaire we surveyed members of a consumer panel and (parents of) patients with Pompe disease in 2010. The study was approved by the institutional review boards of the Erasmus MC University Medical Center and the VU University Medical Center.

### Study population

The Dutch Health Care Consumer Panel is maintained by the Netherlands Institute for Health Services Research (NIVEL) [38]. The panel consists of Dutch individuals over 18 who may be members for three years. Members are recruited using address files purchased from an address file supplier. All 1500 panel members were invited for this study. They were offered the standard incentive of a chance to win a 15 Euro gift certificate.

The Dutch Association for Neuromuscular Diseases (VSN) offers membership to patients, their family members and other associates over 18, including survivors of deceased patients. 100 members (associated) with Pompe disease were eligible for this study. Multiple associates for a single patient were allowed but survivors of deceased patients were excluded.

### Study size

Study size was based on the convenience of a 1500-member consumer panel and on maximal ascertainment of the Pompe (associated) population in the Netherlands.

### Questionnaire overview

Since the survey dealt with a rare disease and a health service, neonatal screening, which gets little publicity, it was necessary to inform participants about the state of the art before asking their opinion. The questionnaire therefore started with illustrated background information on Pompe disease and neonatal screening, including a flow chart for screening and follow-up. To ensure validity of the survey, comprehension was tested and used as an inclusion criterion. The questionnaire also contained vignettes describing three outcomes of a positive neonatal screening test for Pompe disease: classic infantile disease, a false positive result, and early detection of (probable) late-onset disease. The expected incidence of false-positives was calculated by multiplying the hypothetical recall rate from the Austrian pilot study [16] with the number of annual births in the Netherlands. Additional files show an English translation of the original Dutch cover letter [Additional file 1] and the questionnaire used to survey (parents of) patients [Additional file 2]. The questionnaire used for the consumer panel (not shown) had a slightly different lay-out and omitted demographic items which were already known.

### Questionnaire development and pre-test

The questionnaire was meant to be understood by people who have completed secondary education, i.e. at least pre-vocational secondary school. The background description of Pompe disease was based on lay brochures of the Dutch Association for Neuromuscular Diseases but also included additional information. The legibility standard recommended by the Netherlands Central Committee on Research Involving Human Subjects was followed [39].

The questionnaire was pre-tested in 2 phases, with the aim of trouble-shooting [40]. Readers included 12 non-specialists, a patient with late-onset Pompe disease and a professional communication specialist. It took up to 30 minutes to complete the questionnaire.

### Measures and scoring

A supplementary table summarizes the measures used in the questionnaire [Additional file 3]. Comprehension questions were scored as correct or other (incorrect or 'don't know'). A threshold for sufficient comprehension was set at ≥ 3 out of 4 correct answers, including a correct answer to the question on the discriminatory power of the heel stick screening. (Additional file 3, item 4)

Scaled items were scored 1 to 3 or 1 to 5

Educational level was grouped into 3 categories: low (through primary or prevocational secondary school), middle (secondary or vocational school), and high (technical or academic university).

The 2 consumer panel members who reported Pompe disease (in the family) were analyzed together with all the patient organisation members.

The decisive reason (not) to use screening (items 20, 22) was considered valid only if the respondent had correctly followed the flow from item 18 [see Additional file 3].

Ethnicity was coded as Dutch, other Western or non-Western by the following algorithm: by country of birth if not the Netherlands, if the Netherlands then by country of birth of mother, if the subject and mother were both born in the Netherlands then by country of father [41].

Acceptability of the questionnaire was categorized for non-responders who supplied a reason for nonresponse as "advanced age or no children", "too difficult" or "other".

### Data collection and handling

Questionnaires were sent to members of the consumer panel and patient organisation in February 2010. Three weeks later a reminder was sent to non-responders. Questionnaires were processed if they were returned within 3 weeks by the consumer panel or within about 6 weeks by patient organisation members.

Data of the consumer panel was entered manually at NIVEL and linked with previously collected demographic data including non-responder panel members. These anonymous databases were then transferred to the VU University Medical Center. The two consumer panel members who reported having Pompe disease (in the family) were not contacted for verification.

Anonymous data of members of the Dutch Association for Neuromuscular Diseases was entered manually in SPSS at the VU University Medical Center. Logical checks were performed and a 10% sample of the entered data was checked by an independent reader, who found no errors in data entry.

### Efforts to address bias

The composition of the consumer panel is meant to reflect the demographics of the Dutch population, [38] but responders to the questionnaire and the subgroup which demonstrated sufficient comprehension cannot be assumed to be a random sample. Therefore, for analyses where panel opinion was extrapolated to the Dutch population, direct standardization was performed for age and gender, or for educational level. Weights for educational level were calculated with data from Statistics Netherlands [42]. Weights for age and gender were supplied by NIVEL, based on 2009 data from Statistics Netherlands. Non-response was analyzed by educational level and from self-reported reasons for non-response.

### Measures not analysed

Questions on the most important advantage and disadvantage of screening for classic infantile Pompe (items 5 and 6) were put in the questionnaire to provoke thought but were not intended for analysis, because this scenario does not challenge ethical principles of current screening criteria. (These items were in fact used in a post-hoc subgroup analysis, see Qualitative analysis.) It was not possible to meaningfully test the relationship between age and attitude to screening, due to low representation of certain age categories, notably the age category corresponding to young parenthood. Non-Dutch ethnicity was strongly underrepresented in the study population (see [43]); therefore the relationship between ethnicity and attitude to screening was not analyzed. In practice, people with Pompe disease (in the family) would have access to clinical genetic services before a new pregnancy and therefore it is of limited relevance to explore their hypothetical use of screening. For this reason Pompe status was not included in regression analysis of use of screening nor was the decisive moral reason for (not) using screening analyzed for this group; however, a raw figure for hypothetical use of screening is reported.

### Qualitative analysis

In a post-hoc subgroup analysis of opponents to a government offer of screening, free-text responses on the most important advantage and disadvantage of screening in the scenario of classic infantile Pompe disease (items 5 and 6) were analyzed to see if informants had mentioned any merits for screening in the classic infantile scenario. Open coding was done, by one author (SSW) [44].

### Statistical methods

Statistical analysis was done with SPSS for Windows (version 15.0.1, SPSS Inc., Chicago, IL.)

Differences in proportion were tested with Pearson's chi square or Fisher's exact test if any cells had an expected count < 5. Continuous variables were not normally distributed and groups were compared with the Mann Whitney Wilcoxon U-test. Ordinal variables from scaled items are reported as means. For ordinal, including scaled, items differences between groups were tested with the chi squared test for trend; exact 2-sided significance is reported if any cells had an expected count < 5.

95% confidence intervals for single proportions and for differences of proportions were calculated with the software program CIA (Confidence Interval Analysis) with the accurate Wilson-type methods described by Newcombe and Altman [45]. 95% confidence intervals for directly standardized proportions were calculated with CIA by the method described by Morris and Gardner [46].

In univariate logistic regression significance of determinants was interpreted from the Wald statistic. Multiple logistic regression was done by stepwise entering of variables to assess the combined effects of several determinants. Possible interaction between determinants was examined by adding product terms.

If ≥ 5% of valid cases were missing for any item, they were explored for non-randomness of the main demographic variables (gender, level of education, and where relevant Pompe status). Missing data were not analysed in regression analyses.

## Results and discussion

### Response and inclusion

The response rate was 51% for consumer panel members and 59% for patient association members (Table 1). Since basic demographics were available for the entire consumer panel, it could be determined that responders were more likely to have middle and higher education than non-responders (p = 0.01, chi squared test for trend). The most common self-reported reasons for non-response in the consumer panel (n = 117) were advanced age or not having children (22%) and difficulty of the questionnaire (18%).

**Table 1:** Response and inclusion

	Patients' organisation		Consumer panel	
**Response**				

questionnaires mailed	100		1500	

questionnaires filled in (response rate)*^a^*	59	(59%)	757	(51%)

**Pompe disease (in family)**				

Yes	59		2*^b^*	

No	0		750	

item missing	0		5*^c^*	

	**Pompe disease (in family)** (n = 61)		**neutral group** (n = 750)	

**Sufficient knowledge score**				

	58	95%	555	74%

*^a ^*Not counting 5 members of the patients' organisation and 117 of the consumer panel who only provided their reason for non-response

*^b ^*Not verified. One of these responders was later excluded due to insufficient knowledge score.

*^c ^*Excluded from analysis

Two consumer panel members reported Pompe disease (in the family). We cannot exclude that they were also members of the patient organisation, but it is unlikely that they would have filled out the survey twice. In all following analyses, people with Pompe disease (in the family) are classified together and compared to the remaining consumer panel members, redefined as the neutral group (Table 1 lower panel).

After presenting background information comprehension was tested. Based on the results of the pre-test, it was expected that most people with middle or higher education would pass the threshold (see Methods). 74% of the neutral group and 95% of people with Pompe (in the family) demonstrated sufficient comprehension. The comprehension criterion led to a shift of distribution of educational level in the neutral group, towards middle and higher education (p < 0.001, chi squared test for trend).

### Characteristics of study population

Table 2 shows demographic characteristics of the neutral group (n = 555) and the group with Pompe disease (in the family) (n = 58). The neutral group was slightly older than the Pompe group (median 58 versus 52.5, p = 0.002, Mann Whitney U). Gender, educational level, and ethnicity were similarly distributed in both groups. 19% of the neutral group reported having a genetic disease (in the family).

**Table 2:** Characteristics of study population^a^

	Pompe (in family) n = 58	Neutral group n = 555	p
**median age (range)*^b, c^***	52.5 (31-74)	58 (21-91)	0.002

**gender: female**	67%	62%	0.401

**genetic disease in family*^d^***	NA	19%	NA

**educational level*^e^***			0.694

low	21%	18%	

middle	45%	50%	

high	35%	33%	

**ethnicity*^f, g^***			0.565

Dutch	97%	93%	

Other Western country	3%	7%	

Non-Western country	0%	1%	

*^a^*Pearson chi square except where otherwise indicated

*^b^*1 missing

*^c^*Mann-Whitney U

*^d ^*missing; NA = not analyzed

*^e^*16 missing

*^f ^*2 missing

*^g ^*Fisher's exact

### Overall acceptability of screening from public health- and users' perspectives

Acceptability of screening from the public health point of view was measured in two ways: whether the government should offer screening and acceptability of rates of unintended outcomes. 87% of the neutral group and 88% of the Pompe group felt that neonatal screening should be offered for Pompe disease (12 missing; 95% CI of difference -10 to 7%). When the results of the neutral group were standardised to demographics of the Dutch population, a very similar proportion for support was calculated (shown in a supplementary table) [Additional file 4].

72% of the neutral group and 74% of the Pompe group found the expected occurrence of false positives, 60 to 100 per year, acceptable (9 missing; 95% CI of difference -11 to12%). 80% of the neutral group and 86% of the Pompe group found it acceptable that 3 to 5 cases of (probable) late-onset Pompe would be detected annually (14 missing; 95% CI of difference -5 to 14).

The user's perspective on neonatal screening is primarily relevant for the neutral group, since people with Pompe disease (in the family) would have access to other, clinical genetic services before a pregnancy. 87% of the neutral group said they would probably make use of an offer of neonatal screening (18 missing; 95% CI 83 to 89%). The standardized proportion for the Dutch population was very similar, though with a broader confidence interval (shown in a supplementary table) [Additional file 4]. For the sake of completeness it is reported that of respondents with Pompe disease (in the family), 88% (2 missing; 95% CI 76 to 94%) said they would use hypothetically use neonatal screening for their disease.

Next, demographic determinants were explored for the two main outcome measures of support for screening. For the question whether screening should be offered by the government, univariate analysis showed that educational level was a significant predictor for opinion (Table 3A; p < 0.001). The most highly educated group was less likely to favor the offer of screening than the least educated group (OR 0.22, 95% CI 0.09 to 0.53). Multivariate analysis was done to adjust for possible confounding. To limit the number of interaction terms which would have to be tested, lower and middle educational level were hereby combined in one category. Multivariate analysis showed similar odds ratios as univariate analysis (Table 3B, OR 0.29, 95% CI 0.18 to 0.49, p < 0.001), confirming that people with a high level of education were less likely to approve a government offer of screening than people with a lower and middle level of education. Having Pompe disease (in the family) or gender did not explain approval for a government offer of screening.

**Table 3:** Approval of offer of screening by government

	OR	95% CI	p
A. univariate			

**education***^a^*			< 0.001

low	1		

middle	0.64	0.25-1.60	

high	0.22	0.09-0.53	

**gender***^b^*			0.689

male	1		

female	0.90	0.55-1.49	

**Pompe in family*^c^***			0.648

no	1		

yes	1.06	0.46-2.43	

B. multivariate*^d^*			

**education**			< 0.001

low and middle	1		

high	0.29	0.18-0.49	

**gender**			0.290

male	1		

female	0.75	0.45-1.27	

**Pompe in family**			0.777

no	1		

yes	1.13	0.48-2.64	

*^a^*28 missing

*^b^*12 missing

*^c^*12 missing

*^d^*28 missing; odds ratios after adjustment for educational level, gender and Pompe status

As stated earlier, the hypothetical use of screening was relevant only in the neutral group. Univariate analysis showed that use of screening was predicted by educational level (Table 4A; p = 0.001). The most highly educated group was less likely to use screening (OR 0.31, 95% CI 0.13 to 0.73) than the least educated group. There was an indication that women were less likely to use screening than men (OR 0.61, 95% CI 0.36 to 1.05). For multivariate analysis, categories of educational level were combined as described above. Multivariate analysis confirmed that people with a high level of education were less likely to use screening than people with a lower and middle level of education (Table 4B; OR 0.36, 95% CI 0.21 to 0.61, p < 0.001) and suggested that women might be less likely to use screening than men (OR 0.54, 95% CI 0.31 to 0.94, p = 0.029).

**Table 4:** Probable use of screening by the general public

	OR	95% CI	p
A. univariate			

**education***^a^*			0.001

low	1		

middle	0.73	0.31-1.75	

high	0.31	0.13-0.73	

**gender***^b^*			0.075

male	1		

female	0.61	0.36-1.05	

			

B. multivariate*^c^*			

**education**			**<**0.001

low and middle	1		

high	0.36	0.21-0.61	

**gender**			0.029

male	1		

female	0.54	0.31-0.94	

*^a^*34 missing

*^b^*18 missing

*^c^*34 missing; odds ratios after adjustment for educational level and gender

### Valuation of benefits and harms of unintended screening outcomes

False positives and detection of a predisposition for late-onset disease are unintended outcomes of neonatal screening. Benefits and harms of these outcomes, in the context of neonatal screening for Pompe disease, were addressed from the parents' views on the childs' perspective, including a temporal dimension, and the parent.

Both the neutral group and (parents of) Pompe patients saw benefit for *children*, both from a short-term and a lifetime perspective, regardless of whether the outcome of screening was a false positive test or early detection of (probable) late-onset disease (Table 5). Notably, respondents with Pompe disease (in the family) saw more benefit in early detection of late-onset disease than did the neutral group, when considering a child's lifetime perspective (p = 0.017). While the neutral group felt that a child diagnosed early with (probable) late-onset Pompe disease would suffer some harm due to screening, responders with Pompe (in the family) expected significantly less harm for such a child (p = 0.011).

**Table 5:** Valuation of benefits and harms

Child's perspective				Parents' perspective			
	**Pompe*^a^***	**neutral**	**p*^b^***		**Pompe**	**neutral**	**p**

**false positive scenario**							

effect on child 1^st ^year*^c^*	3.47	3.29	0.205	harm to parents	2.05	1.96	0.371

effect on child lifetime*^c^*	3.60	3.50	0.502				

**late-onset scenario**							

effect on child 1^st ^year*^c^*	3.57	3.44	0.444	harm to parents	1.93	1.73	**0.029**

effect on child lifetime	3.93	3.47	**0.017**				

harm to child*^d^*	2.41	2.18	**0.011**				

*^a ^*mean scores; measures of effect scaled 1-5, measures of harm scaled 1-3, with harm at the low end and benefit at the high end of each scale. Items in this table had between 4 and 9 missing values

*^b ^*chi square test for trend

*^c ^*exact chi square test for trend

*^d ^*'(e.g. discrimination)'

Both groups of respondents felt that *parents *confronted with a false positive screening test would suffer a moderate amount of harm (Table 5). The neutral group valued early detection of (probable) late-onset Pompe as more than moderately harmful for parents, while responders with Pompe (in the family) expected significantly less harm for such parents (p = 0.029).

### Moral reasoning

**Figure 1:**
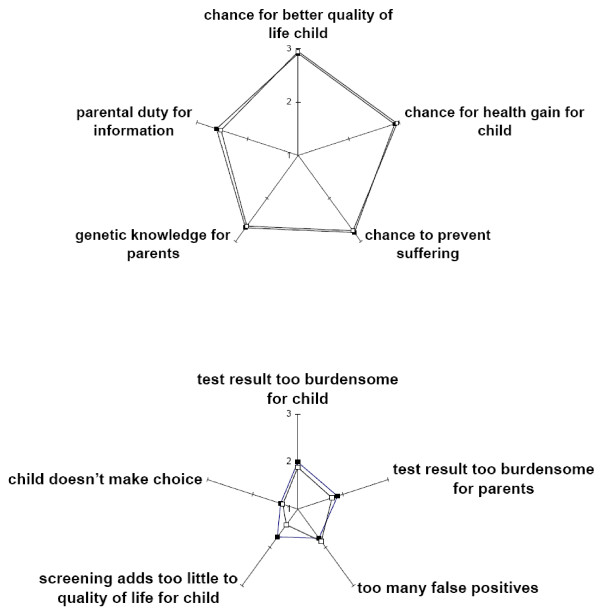
**Valuation of various moral reasons to screen (or not to screen): comparison of neutral and Pompe groups**. Top: importance of reasons to screen^1^. Bottom: Importance of reasons not to screen^2^. Open squares = Pompe group, solid squares = neutral group. ^1^Mean scores of 3-point scale, starting at 1 'unimportant'. Items in top figure had between 10 and 13 missing values. P values of exact chi square test for trend: clockwise, starting at chance for better quality of life child: 0.409, 0.304, 0.665, 0.624, 0.317. ^2^Items in bottom figure had 18 to 27 missing values. P values of (*exact) chi square test for trend: clockwise, starting at test result too burdensome for child: 0.290, 0.228, 0.410, **0.003**, 0.661*.

Regarding autonomy of the child, the responder groups showed similar acceptance of the fact that the child would not make its own choice to be screened for a late onset disease: 89% did not mind in the neutral group and 90% in the Pompe group (9 missing, 95% CI of difference -7 to 10%). Overall, respondents found reasons for screening more important than reasons against screening (Figure 1). The group with Pompe (in the family) attached less importance to the objection that screening adds too little to children's quality of life than did the neutral group (p = 0.003, chi squared test for trend).

**Figure 2:**
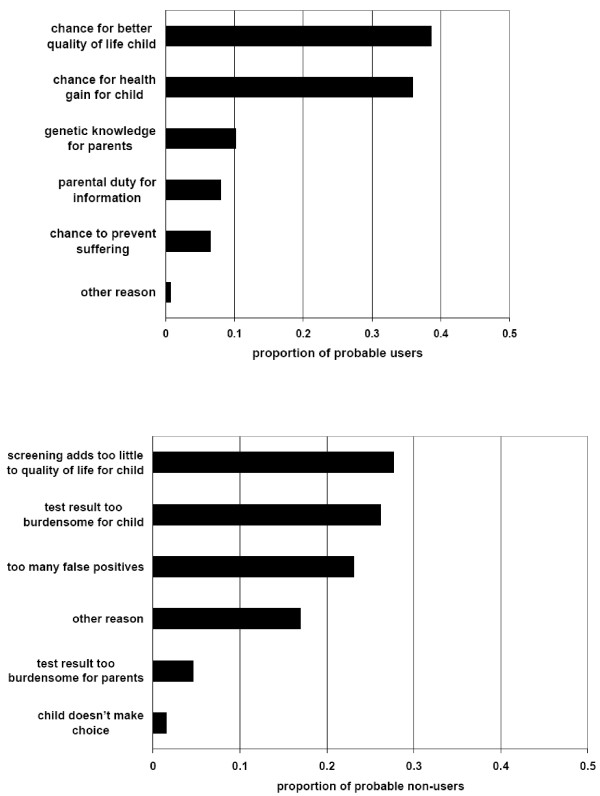
**Decisive moral reason to use (or not to use) screening by neutral group**. Top: probable users of screening^1^. Bottom: probable non-users of screening^2^. See text for missing value analyses. ^1^Moral reasons given by 412 of 465 probable users. ^2^Moral reasons given by 65 of 72 probable non-users.

For the neutral group the questionnaire tried to force a choice for the decisive reason why one would probably (not) use screening (Figure 2). Among probable users of screening the most commonly reported, decisive reasons to use screening were chance for a better quality of life for the child and chance of health gain for the child (Figure 2, top). Analysis of missing values (53 of 465 potential informants, 11%) showed that the lower the educational level, the likelier it was that the respondent did not report a decisive reason to use screening (p = 0.006, chi square test for trend). Among probable non-users of screening, the most commonly reported reason was that screening adds insufficient quality of life for children, followed closely by other reasons (Figure 2, bottom). Analysis of missing values (7 of 72 potential informants, 10%) did not reveal any patterns.

Some respondents formulated their own decisive reason for and/or against using screening (Figure 2, 'other reason'). Some (not shown) were very similar to the ten reasons formulated in the survey. An original reason in favor of screening would be relief if the screening were negative (1×). Original reasons against screening were the absence of Pompe disease in the family (6×), fatalism/reluctance to treat (3×) and low disease frequency (1×). The first category suggests that despite a 'sufficient' knowledge score, some respondents had not grasped the introductory information on risk of recessive inheritance.

### Opponents of screening: exploration of motivation

It was expected that people who opposed a government offer of screening would at least see some merit in early detection of classic infantile disease, when considering the individual child's interest. This hypothesis was explored through post-hoc analysis of selected responses to the vignette on a child with classic infantile disease. Among the 76 opponents of a government offer of screening, 64 did mention a benefit in the case of classic infantile disease, while 10 wrote 'none' or formulated a harm in the space reserved for benefits (2 missing).

## Discussion

The impetus for this study was that several jurisdictions are considering the expansion of neonatal screening to include treatable lysosomal storage disorders. Sometimes patient advocates have promoted the expansion of neonatal screening for particular conditions, while evidence-based reviews by professional experts have been more hesitant [47]. Pompe disease is of special interest because it exemplifies disorders where screening will flag a population of diverse phenotypes. The opinion of citizens is generally unknown. To our knowledge this is the first quantitative study which measured the general public's opinion about neonatal screening for a specific disorder and directly compared it to the opinion of (parents of) patients with that particular disorder.

This study found evidence for rather high support for neonatal screening for Pompe disease in the general public, as measured among consumer panel members who had been effectively informed prior to completing a questionnaire. Their support was consistent, whether questions were framed from the public health perspective (should Pompe disease be added to the neonatal screening program?) or the personal perspective (would you probably use the screening?), and it was confirmed after standardization for demographics of the Dutch population. The expected annual numbers of false positives and (probable) late-onset cases were acceptable to most respondents in the consumer panel. Furthermore, balancing benefits and harms within these two unsought screening outcomes came out on the side of benefit for the child, both in the short- and long term. Moderate harm was expected for parents.

Public opinion as measured in this study can be compared to policies formulated by experts. By far the most common decisive reasons to use screening in this study were the chance for a better quality of life of the child and the chance for health gain. These preferences fit fairly well with the criteria for neonatal screening as formulated by expert advisory boards. For example, the Health Council of the Netherlands formulated the main goal of neonatal screening as prevention of health damage, while it also recognized other advantages though according them lesser weight, e.g. faster diagnosis and better care [48]. In the U.S.A. the Secretary's Advisory Committee on Heritable Disorders in Newborns and Children uses a very similar prioritization [49].

Most informants in this study were not concerned about lack of autonomy of a child being screened for late onset disease. This may be due, at least in part, to the fact that so-called late-onset Pompe disease may manifest in childhood. Among the portion of the general public who would not use screening, the most common decisive reason was concern that a test outcome of 'possibly late-onset Pompe disease' is too burdensome for a growing child, which relates to the ethical principle of avoiding harm. It is difficult to relate this finding to current policy, because *screening *children for broad phenotype conditions is not addressed in guidelines such as the ESHG guideline for genetic testing of children [50] or guidelines for genetic screening for chronic conditions [51]. Notably, testing of asymptomatic children is discouraged unless it has consequences for preventive, medical actions during childhood.

This study had complex findings on whether (parents of) patients with Pompe disease differ from the general public in their support for neonatal screening for their disease. On the one hand (parents of) patients did not exceed the position of the general public when considering neonatal screening from a public health- or population perspective (approval of a government offer of screening, acceptability of rates of false positives and late-onset detection). On the other hand, (parents of) Pompe patients were more supportive of early detection of late-onset disease than the general public; they expected more benefit for such children on a lifetime scale and less harm for their parents. Moral reasoning around screening was similar for the general public and (parents of) patients, with one exception. (Parents of) patients were less concerned than the general public that screening might not improve children's quality of life sufficiently to justify screening. Taken together, these results show that (parents of) patients are as sensitive as the general public to most concerns around neonatal screening for Pompe disease, but (parents of) patients expect more benefit from early detection of late-onset disease than the general public.

Univariate regression analysis showed that educational level explained approval of screening. A post-hoc multivariate analysis, in which some categories were combined, showed that independent of Pompe status, people with high educational level were more likely to be reserved about screening than people with middle or lower education. Further studies should try to confirm this finding, which may be related to the phenomenon that lower educational level is associated with approval for expanding neonatal screening to include untreatable diseases [35]. Regression analyses also suggested that women might be more likely to be reluctant to use screening than men, but the broad confidence interval does not support a strong effect of gender.

This study has several limitations. First, standardization was only done for age and gender or educational level, while other demographic variables may also explain attitude towards neonatal screening for Pompe disease. For the group familiar with Pompe disease, this study did not differentiate between parents and patients, who might well have distinctive perspectives. Next, selection bias may have occurred against the least educated stratum of the targeted population, i.e. people with prevocational secondary education who were classified within lower education. The questionnaire was not pre-tested on readers from this stratum. In the consumer panel, lower education was a risk factor for non-response, for failing the comprehension quiz and for skipping the question on decisive reason to use screening. It is not clear whether lower education also played a role in non-response for the Pompe group. Since the comprehension cut-off used for inclusion excluded a fair proportion (26%) of consumer panel members from analysis, it is noted that over 90% of the excluded group supported an offer of screening and would probably use screening (5 to 8% missing). This suggests that more lenient inclusion criteria would not have changed the study's main conclusions.

Although comprehension was an inclusion criterion, there was some evidence of information bias. First, the fact that some respondents from the general public would decline screening because Pompe does not occur in their family shows that the introductory information did not always succeed in conveying the risk of recessive inheritance. Misconceptions in this area are common, and the written information provided in this study was not followed up by face-to-face discussion or counselling. Second, readers' comments in the pre-test phase suggested confusion with the question "What do you think is the net effect of the screening on this child in its first year of life?" on the late-onset scenario. Responders were unsure which specific benefits and harms they should be weighing, or they found it unacceptable to explicitly offset harms and benefits. Despite possibly weak validity, the question was retained because it created a temporal contrast with the following question, which addressed the life-time perspective.

## Conclusions

The rather high public support measured in the Netherlands for neonatal screening for Pompe disease may be shared by citizens of other countries with a similar level of health care. Yet in other countries too some people, including a portion of the patient community, may have reservations about adding this condition to a state-run public health program. Results of this study suggest that it would be challenging to effectively inform less-well educated parents about the opportunities and risks of neonatal screening for a broad-phenotype condition like Pompe disease. This is especially important for jurisdictions like the Netherlands, which aim for informed consent from parents prior to neonatal screening. Yet regardless of whether screening is mandatory, citizens' views are important for policy-makers and legislators who are judging the benefit-to-risk balance of screening for a new condition. Therefore additional studies should be undertaken to confirm which determinants explain the range of public opinion on neonatal screening for a broad-phenotype condition like Pompe disease. Finally, the hesitation expressed by some respondents for screening for a broad phenotype at birth may be construed as encouragement for exploring alternative ways to shorten the diagnostic delay of Pompe disease.

## Abbreviations

CRIM: Cross-reactive immunological material; NIVEL: Netherlands Institute for Health Services Research; VSN: Dutch Association for Neuromuscular Diseases.

## Competing interests

Salaries of SSW, TR and CGvE were funded by a grant through Top Institute Pharma, which was financially supported by Genzyme Corporation, the Dutch Health Care Insurance Board (College voor Zorgverzekeringen), Shire Corporation, the Dutch Steering Committee on Orphan Drugs, Erasmus MC University Medical Center, Utrecht University Medical Center, and the Academic Medical Center at the University of Amsterdam. The corporate sponsors of this research played no role in the design of the study, review and interpretation of data, or preparation or approval of the manuscript; they only reviewed the manuscript for intellectual property issues.

SSW has a part-time appointment as policy officer at the Dutch Association for Neuromuscular Diseases.

AJJR, ATvdP and MLCH: As of August 2004, AJJR, ATvdP and MLCH have provided consulting services to and have received research funding from Genzyme Corporation, Cambridge, MA, USA, under an agreement between Genzyme Corporation and Erasmus MC. Under this agreement, Erasmus MC and inventors for the method of treatment of Pompe disease by enzyme replacement therapy are entitled to royalty payments.

MCC, WJD, and PJK have nothing to disclose.

## Authors' contributions

MLCH and SSW conceived of the study. MCC, WJD, CGvE, AJJR, TR and ATvdP participated in its design. SSW and PJK performed the statistical analyses. SSW analysed the data and drafted the manuscript. All authors helped critically revise the manuscript and approved the final version.

## Supplementary Material

Additional file 1 **Cover letter to questionnaire**. English translation of the original cover letter to members of the patients' organization. Click here for file

Additional file 2 **Questionnaire**. English translation of the original questionnaire sent to members of the patients' organization. Click here for file

Additional file 3 **Measures**. Table of measures in the questionnaire. Click here for file

Additional file 4 **Estimated support for screening in the Dutch population**. Standardized proportions for approval of screening and probable use of screening. Click here for file
