# Supplementary material for: Public support for neonatal screening for Pompe disease, a broad-phenotype condition
Source: Orphanet J Rare Dis. 2012 Mar 14;7:15. doi: 10.1186/1750-1172-7-15 (PMC3351372; doi:10.1186/1750-1172-7-15)
Supplement: Additional file 2 — Questionnaire. English translation of the original questionnaire sent to members of the patients' organization. [file 1750-1172-7-15-S2.PDF]

VU medisch centrum

Erasmus MC
Universitair Medisch Centrum Rotterdam

Survey

Benefits and risks of heel-stick screening of newborns for Pompe disease

translation

Background information and questions……………………………….p. 1-5
Scenarios and questions……………………………………………..p. 6-13

Some difficult terms are marked in gray and are explained on the bottom of each page.

Photographs were printed with permission of the persons depicted.

Images are blocked in this open-access publication.

Background information

Cause

The cause of Pompe disease is a lack of an enzyme called alpha-glucosidase. A shortage of alpha-glucosidase leads to a muscle disease.

Heredity

Pompe disease is a hereditary condition. Patients have a defect in the genes (DNA) which take care of alpha-glucosidase production. The father and mother of a patient are usually healthy. They each have one ‘wrong’ gene but also each have one ‘healthy’ gene for alpha-glucosidase. This is called carriership.
If two carriers have a child, every pregnancy has a risk of 25% that the child will have Pompe disease.

Various forms of the disease

Pompe disease has various forms. There is an early-onset, rapidly progressive form. This is called the classic infantile form of Pompe disease. All other forms of Pompe disease are included in the umbrella term ‘later forms’.

Characteristics and treatment of the classic infantile form

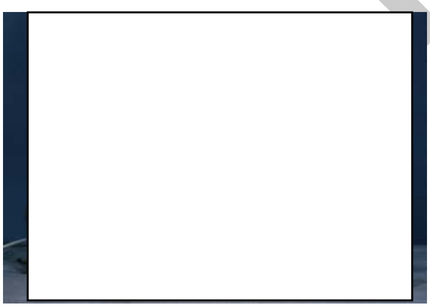

baby with classic infantile Pompe disease

The classic infantile form manifests within a few months after birth. Often there are feeding problems because it is difficult to suck on a bottle. The child is very susceptible to respiratory infections. Disease develops quickly and without treatment babies usually die before the age of one year, due to the consequences of poorly functioning heart- and respiratory muscles.

Alpha-glucosidase: the enzyme which is missing in Pompe disease
Classic infantile: rapidly progressive form of Pompe disease
Later forms: more slowly progressive forms of Pompe disease

nslation

In the last few years enzyme therapy has become available. Enzyme therapy has a beneficial effect on heart function and life expectancy of the children. A few children have reached the age of 10, while their life expectancy without treatment would have been less than a year. However, some of the treated children died or became dependent on assisted respiration. The best results are achieved when treatment is started shortly after birth, before the child's condition has deteriorated too much.

Treatment consists of an infusion of alpha-glucosidase every two weeks. This treatment has to continue lifelong. At present enzyme therapy costs about 300,000 Euros per patient per year. In the Netherlands it is reimbursed.

Characteristics and treatment of later forms of Pompe disease

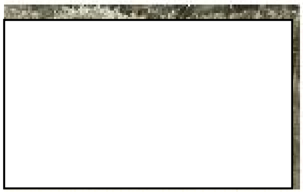
child with later form

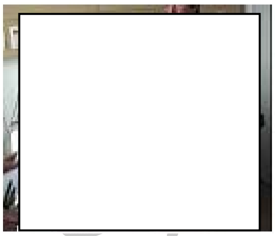
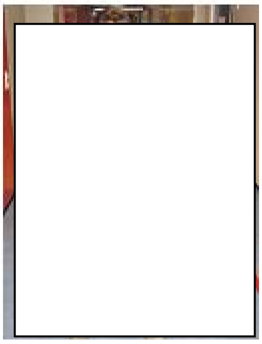
adults with later form

The later forms of Pompe disease can start at any age, in childhood but also as late as adulthood.

People with later forms of Pompe disease have diminished muscle strength, initially especially in their back- and pelvic muscles and later in the trunk. Symptoms start very gradually, but in the end the disease has great impact. Children can develop problems in school with running or push-ups. Problems are usually not acknowledged until people have trouble standing up from a sitting position, start having trouble going up stairs or are unable to get up after a fall. Curvature of the spine (scoliosis) can occur in young people who are still growing. Muscle weakness in skeletal muscles gradually increases so that eventually people are unable to independently stand, walk, sit or turn over in bed.

Alpha-glucosidase: the enzyme which is missing in Pompe disease
Classic infantile: rapidly progressive form of Pompe disease
Later forms: more slowly progressive forms of Pompe disease

Many patients’ respiratory muscles are injured so that ultimately, assisted respiration is often needed. Often problems arise with eating due to difficulties with swallowing and fatigue. Supplementary liquid food or feeding through a gastric tube may be needed to get enough nutrition.
Often patients have had complaints and symptoms of the disease years before they are diagnosed. As of recently enzyme therapy is available. Many people who are now being treated were diagnosed years ago and got worse, before enzyme therapy was available. Enzyme therapy stabilizes or improves muscle strength in many of these people. Lung function seems to stabilize and quality of life improves. Only recently have new patients been able to start enzyme therapy soon after diagnosis. The effect of therapy under these new conditions will become clearer in the next years.

How often does it occur?

There are about 120 patients with Pompe disease in the Netherlands in total. Every year on average 2 babies are born with the classic infantile form, and about 4 babies who will develop a later form of the disease at some point in life.

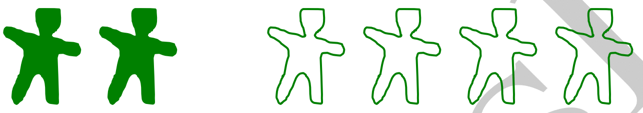

2 classic infantile 4 will develop a later form

Screening of infants for classic infantile Pompe disease in the future?

Screening functions as a primary sorting. From previous research we know that this sorting is not perfect. If heel-stick screening showed that a baby had low alpha-glucosidase activity, there could be several explanations. To know what’s going on, parents would have to take the child to the hospital. There a doctor would examine the child and further blood tests would be needed.
These additional tests would lead to one of the following conclusions:
-the child has classic infantile Pompe disease, or
-there was a technical error in the screening, in other words the child is in fact healthy, or
-the child has low alpha-glucosidase activity but there are no symptoms yet; it might develop a later form of Pompe disease later on.

Taken together, heel-stick screening cannot distinguish between classic infantile and later forms of Pompe disease. Additional tests in the hospital are needed to make the distinction.

translation

Explanation of screening timeline

Newborns in the Netherlands (200,000 per year) are presently screened for various serious, treatable diseases, but not (yet) for Pompe disease. Heel-stick screening for Pompe disease would probably proceed like this.

age	event		
week 0	birth		
week 1	heel stick		
week 1-2	result normal: no Pompe	result suspicious	
week 2		child goes to hospital for physical examination and additional blood tests	
week 2-3	no Pompe (screening was false positive)	classic infantile Pompe	later form Pompe ?
week 3		child starts lifelong enzyme therapy, parents get clinical genetic advice*	From now on repeated follow-up evaluation, parents get clinical genetic advice*
after many years?			symptoms?
			in case of symptoms, lifelong treatment

**Clinical genetic advice: in every subsequent pregnancy there is a 25% risk of recurrence. A clinical genetic counsellor will inform the parents about various options. One of the options is to test the fetus for Pompe disease early in pregnancy using chorionic villus sampling or amniocentesis.*

translation

Questions

The next 4 questions are about the information you just read. We would like to know whether the information was clear.

1. The first symptoms of Pompe disease

- **start in infancy**
- **can appear at any age**
- **don't know**

2. Enzyme therapy is most effective for babies with the classic infantile form of Pompe disease if it is given

- **as soon as possible**
- **before a slowly progressive form of the disease developes**
- **don't know**

3. A man and woman who each have one 'defective'gene for alpha-glucosidase, can have a baby who at a later age will develop symptoms of a late form of Pompe disease.

- **true**
- **false**
- **don't know**

4. Heel-stick screening can, without further testing, distinguish between classic infantile and later forms of Pompe disease.

- **true**
- **false**
- **don't know**

translation

Scenarios and questions

If a newborn had an unfavorable result of heel-stick screening for Pompe disease, the baby would be examined further in a hospital. Various outcomes are possible. In the next section three possible scenarios are presented, each followed by questions.

Scenario 1

The heel-stick screening is suspicious. A doctor in the hospital does additional tests and diagnoses classic infantile Pompe disease. The baby receives enzyme therapy immediately, and every two weeks for the rest of its life. The child will need a lot of care and treatment. The first few years the child will get enzyme therapy as an outpatient in the hospital. If everything goes well, the child can eventually be treated at home. Thanks to the treatment the child has a big chance to survive and develop (to sit, talk and walk). Yet there is also a chance that the child will be handicapped (walking with difficulty, becoming dependent on assisted respiration) or it may die after all.

Summary: in this scenario the diagnosis is ‘classic infantile Pompe disease’

How often? If the heel-stick screening test is introduced in the Netherlands, this situation will probably occur once or twice a year.

1. In this scenario, what are the most important benefits of screening in your opinion?

…………………………………………………………………………………………………

2. What are the most important disadvantages in your opinion?

…………………………………………………………………………………………………

translation

Scenario 2

The heel-stick screening is suspicious. Within a few days the child is extensively examined in the hospital. Afterwards the doctor tells the parents: "I have good news for you. We have examined your child further and she is healthy. It's very unpleasant that you were frightened by the result of the heel-stick screening. It was a so-called 'false positive'. Unfortunately the technique isn't perfect; it happens once in a while."

Summary: in this scenario the screening was false positive; the child is healthy.
How often? If the heel-stick screening test is introduced in the Netherlands, this situation will probably occur 60 to 100 times a year.

translation

3. Is it acceptable to you if this situation occurs 60 to 100 times per year in the Netherlands?

- ☐ **yes**
- ☐ **no**

For the questions below, check the box that represents your opinion.

	very harmful	slightly harmful	neutral	slightly beneficial	very beneficial
4. What do you think is the net effect of the screening on this child in its first year of life?	☐	☐	☐	☐	☐
5. What do you think is the net effect of the screening on this child over its entire life?	☐	☐	☐	☐	☐

6. If you were the parent, how much harm (for example anxiety) would you probably experience?

- ☐ **a lot of harm**
- ☐ **some harm**
- ☐ **a little harm**

Scenario 3

The heel-stick screening is suspicious. Within a few days the child is extensively examined in the hospital. Afterwards the doctor says the following: “Your child is doing well but in her blood we measure very little of the enzyme alpha-glucosidase. It’s likely that at some point your child will develop symptoms which fit with Pompe disease. I can’t say at what age. It can start at any age, but maybe the symptoms will start very late in life or not at all. But the last possibility isn’t very likely. I advise having your child checked in the hospital every half year. In between, if you’re concerned about the health or development of your child, you can always visit sooner. If your child is older and there are still no symptoms, we can do the checkups less often. Right now I don’t advise treatment. If your child develops symptoms of the disease, she can get enzyme therapy.”

Summary: in this scenario the provisional diagnosis is‘ a later form of Pompe disease’.
How often? If the heel-stick screening test is introduced in the Netherlands, this situation will probably occur 3 to 5 times a year.

For the questions below, check the box that represents your opinion.

	**very harmful**	**slightly harmful**	**neutral**	**slightly beneficial**	**very beneficial**
7. What do you think is the net effect of the screening on this child in its first year of life?	☐	☐	☐	☐	☐
8. What do you think is the net effect of the screening on this child over its entire life?	☐	☐	☐	☐	☐

9. If you were the parent, how much harm (for example anxiety) would you probably experience?

- ☐ **a lot of harm**
- ☐ **some harm**
- ☐ **a little harm**

translation

10. How much harm (for example discrimination) do you think the child would experience?

- **a lot of harms**
- **some harm**
- **a little harm**

11. This child itself did not choose to be screened for a late onset disease. How do you feel about this?

- **I mind**
- **I don't mind**

12. Is it acceptable to you if this situation occurs 3 to 5 times per year in the Netherlands?

- **yes**
- **no**

translation

You just answered a set of questions about the three scenarios. Next there are some general questions about heel-stick screening for Pompe disease.

13. All things considered, do you think the government should offer parents of newborns screening for Pompe disease?

☐ **yes**

☐ **no**

14. The tables below contain several reasons for and against screening.
To the right of each reason, put a number indicating how important this reason is to you:

1: not important, 2: somewhat important 3: very important

Reasons to screen	How impor-tant?
Chance of health gain for child	
Genetic knowledge gives parents new choices in further family planning .	
Chance to prevent suffering	
Chance for better quality of life for the child	
Parental duty to gather health information on child	
Other reason, namely... ………………………………………. ……………………………………….	

Reasons not to screen	How impor-tant?
Too many false positives	
The child doesn't make the choice for information about possible late-onset disease	
Screening adds too little to quality of life of children	
An outcome of 'possibly late-onset Pompe disease' is too burdensome for a growing child	
An outcome of 'possibly late-onset Pompe disease' is too burdensome for parents	
Other reason, namely... ………………………………………. ……………………………………….	

translation

15. If you were offered screening of your newborn child for Pompe disease, do you think you would take the offer? (check 1 answer)

probably ☐ (continue with question 16)

probably not ☐ (continue with question 17)

16. What would be your decisive reason for having your child screened?
(underline one answer or fill in the last blank)

Chance of health gain for child
Genetic knowledge gives parents new choices in further family planning
Chance to prevent suffering
Chance for better quality of life for the child
Parental duty to gather health information on child
Other reason, namely ……………………………………………………………….

Skip to question 18.

17. What would be your decisive reason for not having your child screened?
(underline one answer or fill in the last blank)

Too many false positives
The child doesn't make the choice for information about possible late-onset disease
Screening adds too little to quality of life of children
An outcome of 'possibly late-onset Pompe disease' is too burdensome for a growing child
An outcome of 'possibly late-onset Pompe disease' is too burdensome for parents
Other reason, namely ……………………………………………………………….

translation

Items 18-20 are beyond the scope of the current paper.

	Completely agree	Partially agree	neutral	Partially disagree	Completely disagree
18. Useful screening tests should be payed for by the government or through basic health insurance.	☐	☐	☐	☐	☐
19. It's good that private companies offer screening, as long as the quality is guaranteed.	☐	☐	☐	☐	☐

20. If you had to pay to have your newborn screened for Pompe disease, how much would you probably be willing to pay? (check one box)

☐ **nothing** ☐ **maximum 25 euro** ☐ **maximum 250 euro** ☐ **more than 250 euro**

translation

Last, we request some personal information to help us understand the distribution of opinions in the population.

21. How old are you? ....

22. What is your gender? □ **male** □ **female**

23. Do you, or does anybody in your family, have a genetic disease? □**yes** □ **no**

24. Do you, or does anybody in your family, have Pompe disease? □**yes** □ **no**

25. If applicable, what is your religion?...

26. If you skipped any questions in this survey, for what reason?

………………………………………………...

27. What is the highest level of education you have attained? (education completed with a diploma or references of satisfactory completion)

- ❑ no education (primary school: did not finish)
- ❑ primary education (*basisschool, speciaal basisonderwijs*)
- ❑ lower or preparatory vocational training (e.g. *LTS, LEAO, LHNO, VMBO*)
- ❑ middle level secondary education (e.g. *MAVO, (M)ULO, MBO-kort, VMBO-t*)
- ❑ middle level vocational- or apprenticeship training (e.g. *MBO-lang, MTS, MEAO, BOL, BBL, INAS*)
- ❑ higher secondary, or college preparatory education (e.g. *HAVO, VWO, Atheneum, Gymnasium, HBS, MMS*)
- ❑ higher professional education (e.g. *HBO, HTS, HEAO, HBO-V, kandidaats wetenschappelijk onderwijs*)
- ❑ university education
- ❑ other, namely ....…………………………………………………

28. In what country were you born?...

29. In what country was your mother born?..

30. In what country was your father born?..

translation

Thank you for your contribution to this study!
