# Supplementary material for: Public support for neonatal screening for Pompe disease, a broad-phenotype condition
Source: Orphanet J Rare Dis. 2012 Mar 14;7:15. doi: 10.1186/1750-1172-7-15 (PMC3351372; doi:10.1186/1750-1172-7-15)
Supplement: Additional file 3 — Measures. Table of measures in the questionnaire. [file 1750-1172-7-15-S3.PDF]

Table- Questionnaire measures[a]

**concept**				
**scenario/ perspective**	**item**	***original number***	**question**	**answer categories**
comprehension of issues				
	1	*1*	The first symptoms of Pompe disease ...	-start in infancy<br>-can appear at any age<br>-don't know
	2	*2*	For babies with classic infantile Pompe, enzyme replacement therapy is most effective when given...	-as soon as possible<br>-before late-onset disease sets in<br>-don't know
	3	*3*	A couple who each have one "incorrect" gene for alpha-glucosidase can have a baby who will later on in life develop symptoms of a later-onset type of Pompe disease.	true<br>false<br>don't know

	4	*4*	Heel stick screening can, without additional testing, distinguish between classic infantile and later types of Pompe disease.	true<br>false<br>don’t know
acceptability of various screening outcomes				
<u>classic infantile Pompe</u>	5	*1*	In this scenario, what are the most important benefits of screening in your opinion?	Open
	6	*2*	What are the most important disadvantages in your opinion?	open
<u>false positive</u>				
child’s perspective	7	*4*	What do you think is the net effect of the screening on this child in its first year of life?	5-point bipolar scale: very harmful to very beneficial
	8	*5*	What do you think is the net effect of the screening on this child over its entire life?	5-point bipolar scale: very harmful to very beneficial

parents' perspective	9	*6*	If you were the parent, how much harm (for example anxiety) would you probably experience?	3-point scale: a lot of harm to little harm
population perspective	10	*3*	Is it acceptable to you if this situation occurs 60 to 100 times per year in the Netherlands?	yes<br>no
<u>early detection of late-onset Pompe</u>				
child's perspective	11	*7*	What do you think is the net effect of the screening on this child in its first year of life?	5-point bipolar scale: very harmful to very beneficial
	12	*8*	What do you think is the net effect of the screening on this child over its entire life?	5-point bipolar scale: very harmful to very beneficial
	13	*10*	How much harm (for example discrimination) do you think the child would experience?	3-point scale: a lot of harm to little harm
	14	*11*	This child itself did not choose to be screened for a late onset disease. How do you feel about this?	I mind.<br>I don't mind.

parents' perspective	15	*9*	If you were the parent, how much harm (for example anxiety) would you probably experience?	3-point scale: a lot of harm to little harm
population perspective	16	*12*	Is it acceptable to you if this situation occurs 3 to 5 times per year in the Netherlands?	yes<br>no
overall acceptability of screening				
screening as a public health duty	17	*13*	All things considered, do you think the government should offer parents of newborns screening for Pompe disease?	yes<br>no
screening as a personal choice	18	*15*	If you were offered screening of your newborn child for Pompe disease, do you think you would take the offer?	probably<br>probably not

moral reasoning				
valuation of reasons for government offer of screening	19	*14*	• Chance of health gain for child<br>• Genetic knowledge gives parents new choices in further family planning.<br>• Chance to prevent suffering<br>• Chance for better quality of life for the child<br>• Parental duty to gather health information on child<br>• Other reason, namely...[space to specify]	for each reason:<br><br>3-point scale: not important to very important
decisive reason for own use of screening	20[b]	*16*	6 reasons as above	forced choice of 1
valuation of reasons against government offer of screening	21	*14*	• Too many false positives<br>• The child doesn't make the choice for information about possible late-onset disease<br>• Screening adds too little to quality of life of children<br>• An outcome of 'possibly late-onset Pompe disease' is too burdensome for a growing child<br>• An outcome of 'possibly late-onset Pompe disease' is too burdensome for parents<br>• Other reason, namely...[space to specify]	for each reason:<br><br>3-point scale: not important to very important

decisive reason against own use of screening	22[c]	*17*	6 reasons as above	forced choice of 1
Demographics				
	23	*21*	Age	open
	24	*22*	Gender	male<br>female
	25	*23*	Do you, or does anybody in your family have a genetic disease?	yes<br>no
	26	*24*	Do you, or does anybody in your family have Pompe disease?	yes<br>no
	27	*25*	If applicable, what is your religion affiliation?	open
	28	*27*	What is your highest completed level of education?	8 categories + "other"
	29	*28*	In which country were you born?	open
	30	*29*	In which country was your mother born?	open
	31	*30*	In which country was your father born?	open

suitability of questionnaire				
	32	*26*	If you skipped any questions [in the entire questionnaire], can you indicate why?	open

[a]The original order of the questions is shown in Additional file 2 and in italics in column 3.

[b] This item was only to be answered by probable users of screening (item 18).

[c] This item was only to be answered by probably non-users of screening (item 18).
