# Supplementary material for: Public support for neonatal screening for Pompe disease, a broad-phenotype condition
Source: Orphanet J Rare Dis. 2012 Mar 14;7:15. doi: 10.1186/1750-1172-7-15 (PMC3351372; doi:10.1186/1750-1172-7-15)
Supplement: Additional file 4 — Estimated support for screening in the Dutch population. Standardized proportions for approval of screening and probable use of screening. [file 1750-1172-7-15-S4.PDF]

Estimated support for screening in the Dutch population

standardization variables	age and gender		educational level	
	proportion	95% CI	proportion	95% CI
**public health perspective** would approve of government offer of screening	86%[1]	74% to 97%	88%[2]	80% to 97%
**users' perspective** would probably use screening	85%[3]	73% to 98%	88%[4]	79% to 96%

[1]Based on data from consumer panel members with sufficient knowledge, including 1 panel member with Pompe (in the family): n=544
[2]n=528
[3]n=538
[4]n=522
