# Supplementary material for: Public support for neonatal screening for Pompe disease, a broad-phenotype condition
Source: Orphanet J Rare Dis. 2012 Mar 14;7:15. doi: 10.1186/1750-1172-7-15 (PMC3351372; doi:10.1186/1750-1172-7-15)
Supplement: Additional file 1 — Cover letter to questionnaire. English translation of the original cover letter to members of the patients' organization. [file 1750-1172-7-15-S1.PDF]

VU medisch centrum

Erasmus MC
Universitair Medisch Centrum Rotterdam

February 15, 2010

Dear madam/sir,

In the Netherlands and many other countries newborns are screened for several diseases. This is done with a so-called heel stick. Shortly after birth a few drops of blood are taken and sent to a laboratory for testing. Currently newborns in the Netherlands are screened with the heel stick for 17 diseases. In the future screening may be extended to more diseases, for example to Pompe disease. This is a rare neuromuscular disorder.

In the Netherlands we screen for diseases which are serious, treatable, and where prompt treatment directly benefits the child. Screening must not have too many disadvantages, such as high cost or dubious results. In practice, no screening test is perfect. The government carefully considers benefits and harms, before public health authorities offer a new screening test to the population.

The VU Medical Center and Erasmus MC are studying the benefits and harms of heel stick screening for Pompe disease. We are also studying whether there is public support for this screening. In the process we are polling the opinion of people of various ages and backgrounds. You are therefore cordially invited to complete this survey.

What are we asking you to do?
The questionnaire starts with background information, followed by several questions. Together, this part of the survey takes about 15 minutes.
Next you will read three “scenarios”, short descriptions of how screening might turn out. These are followed by a longer set of questions. This part of the survey also takes about 15 minutes. We request that you go through the survey in the order in which it is presented (from beginning to end) and that you answer all the questions.

When you are finished
Please send the completed questionnaire in the return envelope <u>before March 5</u>. You don’t need a stamp.

If you don’t want to fill in the questionnaire
If you decide not to participate in the survey, we invite you to at least reply to questions 21 through 30 on page 13, the last page of the questionnaire. Then you can send in the questionnaire in the return envelope. You don’t need a stamp. Please note, for this study it is important to know whether and why certain groups of people decline to fill in the questionnaire.

translation

What happens with the results?
We will process your answers anonymously. When the study is finished we will write a report. This will be available digitally through the Dutch Association for Neuromuscular Disorders (www.vsn.nl) and the Netherlands Institute for Health Services Research www.nivel.nl/consumentenpanel).

Do you have any questions?
If you want more information about this questionnaire or the study, you can call or send an e-mail to Tessel Rigter, researcher [phone number and e-mail address].

Thank you very much for the time you are taking to contribute to this study!

Yours sincerely,

On behalf of the researchers at the EMGO+ Institute (VU Medical Center) and the Center for Lysosomal and Metabolic Diseases (Erasmus MC):

Drs.Tessel Rigter
Dr. Stephanie Weinreich
Dr.ir. Marloes Hagemans
Prof.dr. Ans van der Ploeg

translation
